# Commentary: Value of prolonged scrotal drainage after penile prosthesis implantation: a multicentre prospective nonrandomized pilot study

**DOI:** 10.1038/s41443-023-00740-2

**Published:** 2023-07-24

**Authors:** Wai Gin Lee, Philippa Ralph, David Ralph

**Affiliations:** Department of Urology, University College London Hospitals, London, W1G 8PH UK

**Keywords:** Surgery, Erectile dysfunction

There is a surgical aphorism that has been repeated time and time again: “you never regret leaving a drain in”. However, does this hold true in contemporary clinical practice? The question of placing a scrotal drain following uncomplicated (“virgin”) inflatable penile prosthesis (IPP) insertion has been debated for decades. Current estimates of the risk for developing haematoma following uncomplicated IPP insertion is around 5% in high volume centres [1]. The risk of haematoma following “complex” IPP are higher. Scrotal haematoma results in slower recovery, more pain and complicate cycling of the device. The pump may also migrate to a less accessible location. The haematoma will eventually drain through the surgical wound leading to skin dehiscence and device infection and/or erosion.

Some surgeons are reluctant to place a closed suction scrotal drain after uncomplicated IPP insertion due to the risk of infection [2]. Bacteria may theoretically migrate along the drain tubing and into the wound bed. The drain may also fracture, and a fragment could be retained in the wound. It is also inconvenient for the patient because they may need to return to the office for the drain to be removed. For the surgeon, placing a drain would incur higher costs and take more surgical time [3].

This study by Osmonov et al. is therefore a welcome addition to the literature [4]. The multicentre prospective non-randomised pilot study compared outcomes following IPP insertion via penoscrotal approach in uncomplicated cases [4]. Patients were divided into 3 groups based on the duration of closed-suction drainage of the scrotum postoperatively. Group 1 (*n* = 114) did not have a drain placed; group 2 (*n* = 114) had a drain placed for 24 h while group 3 (*n* = 117) had a drain placed for 72 h.

The 72 h group had a lower incidence of postoperative scrotal haematoma compared to the 24 h group and the no-drain group. The risk of infection following IPP insertion was the same between those with or without a drain. As expected, developing haematoma at 24 h after surgery was associated with a higher incidence of postoperative infection. This finding gives further comfort to surgeons when placing a closed suction drain that the risk of infection will be no higher. Interestingly, the infection rate in the study was higher than would be expected in a cohort of uncomplicated patients following IPP implantation.

There is a dearth of urology-specific data on the value of a scrotal drain following IPP implantation. Drain output increases with a longer surgical time and the rate of drain output is much higher in the first 12 h after surgery compared to the second 12 h period after surgery (11.0 and 2.5 mL/h respectively) [5]. However, whether a scrotal drain prevents postoperative haematoma could not be determined because the study did not have a control group. The drain volumes reported were also surprisingly high (mean volume 161.1 mL).

A much larger retrospective cohort study reported a postoperative infection rate of 3.3% following IPP insertion [6]. This older study from 2005 also did not have a control group and the devices were not antibiotic- or hydrophilic-coated. The scrotal haematoma rate was 0.7%. The report concluded that the use of a closed-suction drain did not increase the risk of prosthesis infection while minimising the risk of haematoma formation. The other data referenced in the literature are conference abstracts (without peer review) and should be interpreted with caution.

A key unanswered question is how this protocol can be implemented in clinical practice and specifically, how patients can be counselled to accept this. The paper alluded to this conundrum in the last sentence of the discussion. The authors declared that “preoperative counselling and postoperative management of the patients may be heterogenous owing to the multicentric nature of our study”. If compliance within the context of a clinical trial may not have been complete, patients would need to be counselled carefully and understand the benefits of prolonged surgical drainage despite the inconvenience. Many institutions discharge men on the same or following day and they would then need to return for the drain to be removed.

Also, daily drain outputs were not measured in the study. These data may have allowed surgeons to “triage” or predict those who will need 72 h drains and those who may have their drain removed earlier. However, the recent paper by Braun et al. reported that a haematoma requiring surgical drainage tended to occur within 72 h of implantation, despite acceptable postoperative drain outputs within the first 24 h [1]. These findings support the proposal to leave scrotal drains for 72 h, particularly following complex IPP insertion.

In conclusion, the study by Osmonov et al. is an important contribution to the literature on the benefit of a scrotal drain following uncomplicated IPP implantation. The take home message is that a drain is unlikely to increase the risk of infection following surgery, and surgeons should have a low threshold to leave the drain in for longer as it will reduce scrotal haematoma risk. A randomised controlled trial may never eventuate meaning that the results of this study should give further confidence to surgeons contemplating whether to place a scrotal drain.
